# Role of Histone Tails in Structural Stability of the Nucleosome

**DOI:** 10.1371/journal.pcbi.1002279

**Published:** 2011-12-15

**Authors:** Mithun Biswas, Karine Voltz, Jeremy C. Smith, Jörg Langowski

**Affiliations:** 1 Computational Molecular Biophysics, Interdisciplinary Center for Scientific Computing (IWR), University of Heidelberg, Heidelberg, Germany; 2 Biophysics of Macromolecules, German Cancer Research Center (DKFZ), Heidelberg, Germany; 3 University of Tennessee/Oak Ridge National Laboratory Center for Molecular Biophysics, Oak Ridge National Laboratory, Oak Ridge, Tennessee, United States of America; 4 Biophysics of Macromolecules, German Cancer Research Center (DKFZ), Heidelberg, Germany; Centro de Investigación Príncipe Felipe (CIPF), Spain

## Abstract

Histone tails play an important role in nucleosome structure and dynamics. Here we investigate the effect of truncation of histone tails H3, H4, H2A and H2B on nucleosome structure with 100 ns all-atom molecular dynamics simulations. Tail domains of H3 and H2B show propensity of 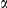-helics formation during the intact nucleosome simulation. On truncation of H4 or H2B tails no structural change occurs in histones. However, H3 or H2A tail truncation results in structural alterations in the histone core domain, and in both the cases the structural change occurs in the H2A
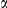
3 domain. We also find that the contacts between the histone H2A C terminal docking domain and surrounding residues are destabilized upon H3 tail truncation. The relation between the present observations and corresponding experiments is discussed.

## Author Summary

Histone tails are the most common sites of post-translational modifications. Tail modifications alter both inter and intra nucleosomal interactions to disrupt the condensed chromatin structure, thereby playing crucial role in gene access. Here we investigated histone tail functions on the stability of a single nucleosome in atomic detail by selectively truncating tail domains in molecular dynamics simulations. Our study revealed that truncation of H3 or H2A tail results in structural alterations in the nucleosome core whereas truncation of H4 or H2B tail does not. A potential role of H2A C terminal tail in regulating nucleosome stability is discussed. Finally, an -helical domain formation was observed in one of the H3 tails and, upon truncation of this tail, structural changes occurred in closely lying histone domains. The correlation between tail-truncation and structural changes likely sheds light on allosteric regulation of nucleosome stability.

## Introduction

Eukaryotic DNA is organized into nucleosomes [1], in which about 150 bp of DNA are wrapped in left-handed superhelical turns around an octameric histone protein complex [2]. The histone octamer has a tripartite structure composed of a 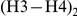 tetramer flanked by two H2A–H2B dimers.

**Figure 1:**
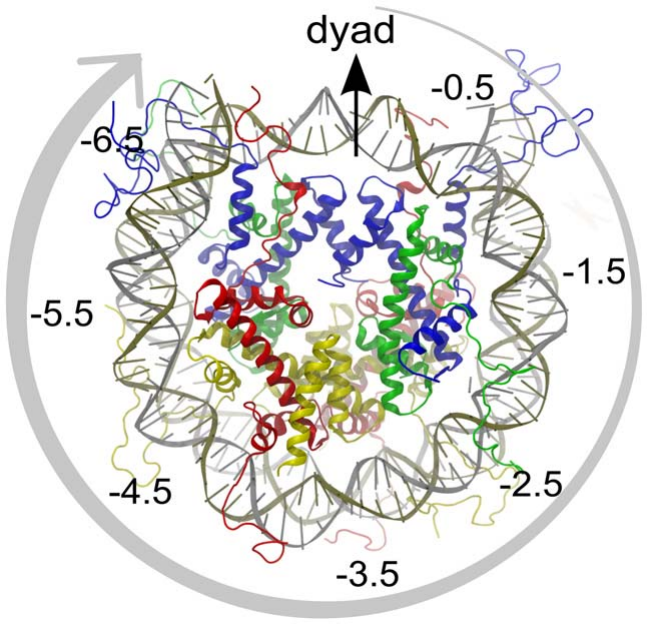
Architecture of the nucleosome core particle (PDB ID 1KX5; [**2**]). The four histone dimers H3, H4, H2A and H2B are colored in blue, green, red and yellow, respectively. The four histone dimers are arranged about a twofold dyad symmetry axis, which also intersects the middle of the DNA fragment. DNA positions around the nucleosome are described by super helical locations, or SHLs, numbered here. The middle of the DNA fragment at the dyad position is referred to as SHL0. Starting from the dyad along the outer wrap of DNA on the nucleosome (shown by an arrow in the figure), each minor groove facing the histone core is denoted by SHL
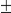
0.5, SHL
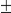
1.5, etc. (positive in one direction, negative in the other).

The 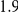 Å resolution structure of the nucleosome core particle revealed interactions between the histone core, histone tails and DNA at atomic detail [2]. In the structure the four histone dimers (two each of H3, H4, H2A and H2B) are arranged about a two-fold dyad symmetry axis, which also intersects with the middle of the DNA fragment. Each of the histone proteins consists of a structured core and a unstructured tail domain. The core domains consist of three 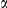-helices (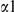, 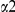 and 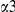), connected by short loops L1 and L2 and are composed mainly of basic residues, except for an acidic patch of H2A near the center of the nucleosome. All four histones have an N-terminal tail domain but only histone H2A has a long C-terminal tail with a large interface with the histone H3–H4 core domains. Positively-charged histone tails make specific interactions with negatively-charged DNA [2]. There are 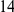 positions on the nucleosomal DNA at which histone residues make contact with DNA by hydrogen bond formation. The positions of DNA around the nucleosome (also referred as super helical locations or SHLs) are often described with respect to the position of the dyad, as shown in Fig. 1.

The nucleosomal organization of eukaryotic chromatin presents a physical barrier to DNA access and also acts as a repository of epigenetic marks controlling chromosomal behavior during different periods of the cell cycle [3]. Chromatin remodelling enzymes can read these epigenetic marks and use ATP to assemble, reposition or evict nucleosomes [4], [5]. Several eukaryotic organisms employ isoforms of histone proteins to regulate DNA genomic access during different periods of the cell cycle [6].

Post-translational modifications of histones play a key role in the regulation of gene access in eukaryotes [7], [8]. The majority of these modifications occur in the N-terminal extensions of the histones in the form of methylation, acetylation or phosphorylation of amino-acid residues [9].

A major challenge in chromatin research is to characterize the effect of tail modifications on nucleosome mobility and stability. Evidence suggests that the modifications may recruit chromatin-binding proteins [10] or may act as a switch between different chromatin states [11]. Mutation or deletion of tail domains has been shown to result in transient unwrapping of DNA near the edge of the nucleosome, variation in the rate of nucleosome sliding on DNA and variation in the rate of H2A–H2B dimer exchange in vitro [12]. Deletion of certain tails also prohibits the formation of condensed chromatin fiber [12], [13].

Truncating the end of the H2A C terminal domain results in a 2.4 fold increase in the nucleosome sliding rate [12]. Also, a number of mutations in the H3–H4 histone fold region that lies close to the H2A C terminal extension, which has a large interface with H3–H4 tetramer, have been found to result in higher nucleosome mobility [12], [14], . Some of these mutations destabilize dimer-tetramer association to the extent that the histone octamer cannot be formed in vitro [16]. The above findings suggest that the destabilization of the H2A C terminal tail may affect nucleosome mobility by altering C terminus-DNA contacts or by modifying histone dimer-tetramer association at the interface with H3/H4. Furthermore, a comparison between wild type and histone variant H2A.Z, an essential histone variant found in all higher eukaryotes with altered histone dimer-tetramer interaction [17], has shown that regions of the H2A.Z sequence essential for biological activity are clustered at the H2A C terminus [18].

Although progress has been made [19], it is not clear how tail modification is related to nucleosomal stability. An atomic level understanding of interactions within the nucleosome that explains experimental findings [12], [20] upon tail truncation is lacking. Here, as a step towards this understanding, we report on a total of 800 ns of all-atom molecular dynamics simulation of intact and tail-truncated nucleosomes and examine the effect of tail truncation on nucleosome structure at atomic detail. We find that both histone H3 and H2A tail truncation destabilize nucleosome structure and that the destabilization involves the same domain of H2A in both cases. We also find that modified interactions at the H2A C terminal interface are related to nucleosome destabilization in a manner that may help explain experimental findings.

## Materials and Methods

**Figure 2:**
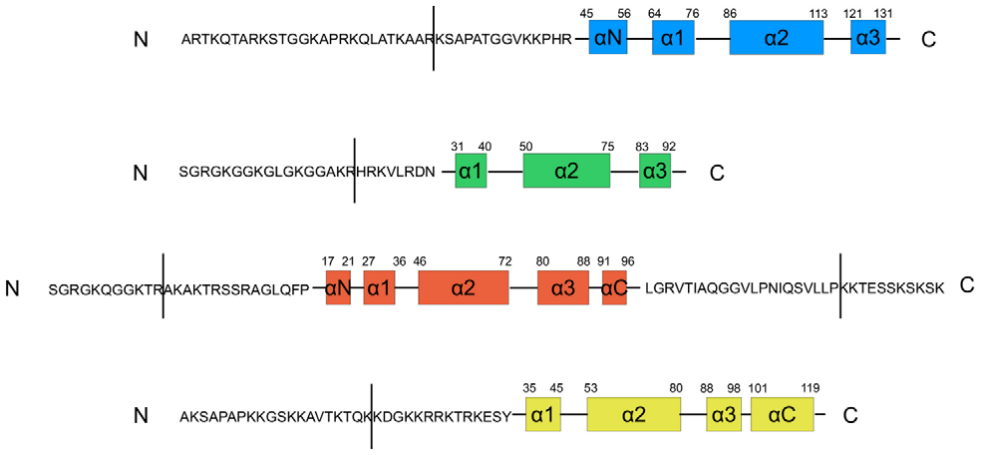
Tail domains of the core histone proteins with positions where they were clipped indicated by lines. In the tail-truncated nucleosome simulations the N-terminal tail domains of H3, H4, H2A and H2B were removed up to residues 26, 17, 11 and 20, respectively. For the H2A tail-truncated simulation the C-terminal residues 118–128 were also removed. Truncation sites for each tail were chosen at known trypsin cleavage sites [44].

Simulations were performed on the intact nucleosome and on tail truncated nucleosomes with each of the four types of histone tail (H3, H4, H2A and H2B) truncated, one at a time. The N-terminal tail domains of H3, H4, H2A and H2B were removed up to residues 26, 17, 11 and 20, respectively, and residues 118–128 were also removed from the C-terminal tail domain of H2A (Fig. 2). Simulations of tail-truncated nucleosomes are denoted by the name of the histone tail removed.

### Molecular Dynamics Simulation Set-up

The starting structure of all molecular dynamics (MD) simulations was taken from the 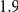 Å resolution crystal structure of the nucleosome core particle (PDB ID: 1KX5) [2]. All simulations used the CHARMM27 force field [21] in the NAMD program [22]. The structures were immersed in a cubic box of TIP3P water molecules and there was at least 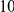 Å of separation between the solute and the edge of the box. The system was then neutralized with 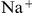 ions and appropriate amount of NaCl was added to keep the systems at 150 mM salt concentration. Each solvated system contains about 200,000 atoms. Periodic boundary conditions were used and the long range electrostatics was treated with the particle mesh Ewald method [23] with a grid size of 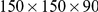 and sixth order interpolation to compute potential and forces between the grid points. For the van der Waals interactions a switching function was applied at 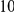 Å and the cut-off was set to 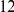 Å. The SHAKE algorithm [24] was used to constrain bonds containing hydrogen atoms. The integration time step was 2 fs and coordinates were saved every 1000 steps during the simulations. The pressure was kept constant at atmospheric pressure at sea level with the Nosé-Hoover Langevin piston pressure control [25], [26] in NAMD. The temperature was maintained at 300 K with a Langevin damping coefficient of 2 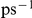.

For each of the simulations, the water molecules and ions were first energy minimized and equilibrated at 300 K for 160 ps with the solute kept fixed. The whole system was then energy minimized for 10000 steps using the conjugate gradient method and keeping the positions of the protein backbone atoms fixed. Harmonic restraints on the backbone atoms were then relaxed stepwise during 30 ps heating and 160 ps equilibration of the whole system.

It was observed that H3 and H2A tail truncation destabilizes the nucleosome structure. To verify these results 2 additional independent simulations of H3 tail-truncated nucleosome and 1 additional simulation of H2A tail-truncated nucleosome were performed. Each of the simulations was 100 ns long, making a total of 8 simulations and 800 ns of combined trajectory. On the Kraken Cray XT5 machine (http://www.nics.tennessee.edu/computing-resources/kraken) generating 1 ns of trajectory took approximately 1700 CPU hours on 504 cores generating about 50 gigabyte of raw data.

### Analysis of Trajectories

The *VMD* [27] and *PyMOL* [28] program was used for visualization of the trajectories and preparation of most of the figures. The root mean square deviation (RMSD) of the trajectories was calculated using the *GROMACS* [29] program. The *3DNA* program [30] was used for the calculation of DNA structural parameters.

The electrostatic potential map was computed using the pmepot plugin in *VMD* with a grid resolution of 1 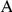 and Ewald factor value of 0.25 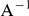.

## Results

### Stability of Nucleosome Structure

**Figure 3:**
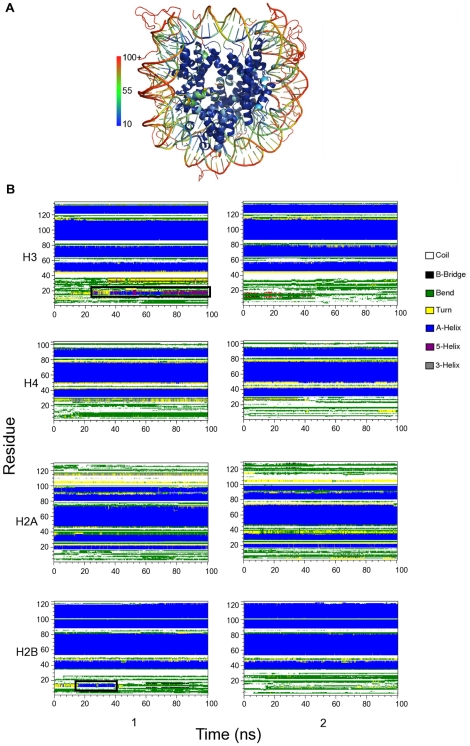
Structural fluctuations in the nucleosome. (A) Temperature factor of the nucleosome in cartoon representation. The atoms are colored as indicated in the scale. (B) Secondary structure of the histone calculated using the *dssp* program [45]. During the simulation amino acids 14–20 of H3 (copy 1) and amino acids 11–15 of H2B (copy 1) show propensity to form 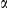-helices.

In the intact nucleosome simulation nucleosomal DNA remains more mobile than the histone fold regions (Fig. 3A) as also observed in the crystal structure [2]. In the histone fold region, amino acids from 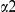 and 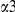 domain of histone H2A show higher mobility. DNA phosphate atom mobility is minimal at histone-DNA contact points and is in good agreement with crystallographic B-factors in Supplementary Fig. S1 (also see Text S1).

In the crystal structure of the nucleosome core particle (1KX5.pdb) histone tails adopt disordered conformations with many amino acids having occupancy zero. In the simulation these tail domains primarily remain bound to DNA with a low degree of ordering. The overall secondary structure conservation in histones is plotted in Fig. 3B which shows that amino acids 14–20 of histone H3 (copy 1) and amino acids 11–15 of histone H2B (copy 1) have propensity to form 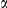-helices.

**Figure 4:**
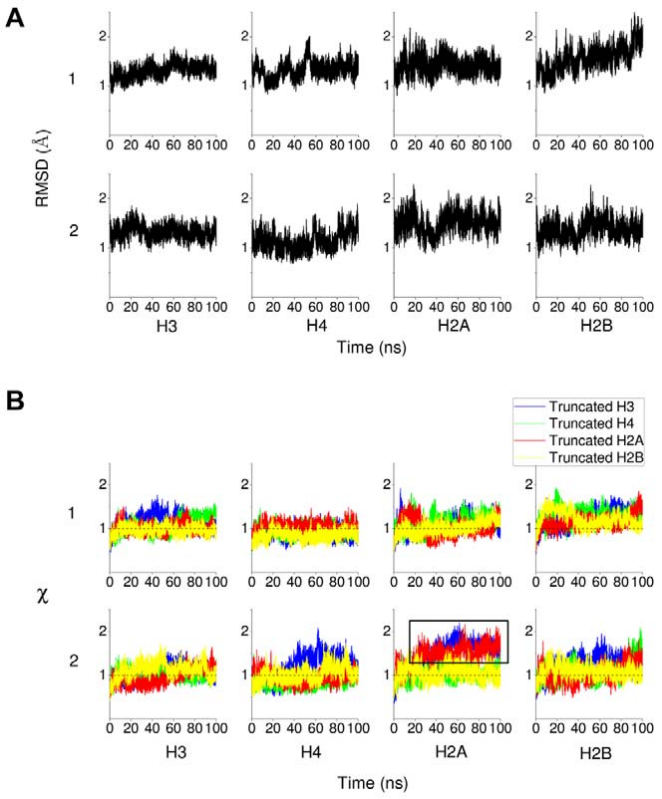
RMSD and order parameter 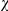
 **of histone monomers H3, H4, H2A and H2B.** (A) RMSDs for each of the two copies, 1 and 2, of the histone monomer backbone (excluding histone tails) versus simulation time for intact nucleosome. The last frame of the equilibration run was chosen as the reference structure. Trajectory frames were reoriented to the reference structure with least square fitting of backbone atoms (excluding tails). (B) Order parameter 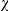 of each of the two copies (numbered 1 and 2) of histone monomers H3, H4, H2A and H2B for the four tail-truncated simulations. The reference structures used for RMSD calculations of the truncated and intact nucleosomes were aligned and had zero RMSD. The dotted lines indicate 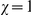.

To examine the overall stability of the histones during the 100 ns intact nucleosome trajectory we calculated the RMSD of each histone (excluding tails) from the equilibrated structure (Fig. 4A). The plot shows that histone core domains stay close to the equilibrated structure during the 100 ns long production simulation.

To quantify the overall effect of tail truncation on the nucleosome structure, we define an order parameter, 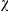, as the ratio of truncated nucleosome root mean square deviation (RMSD) to the intact nucleosome RMSD averaged over the entire trajectory, i.e.
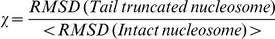


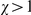
 implies destabilization of a histone domain.

**Figure 5:**
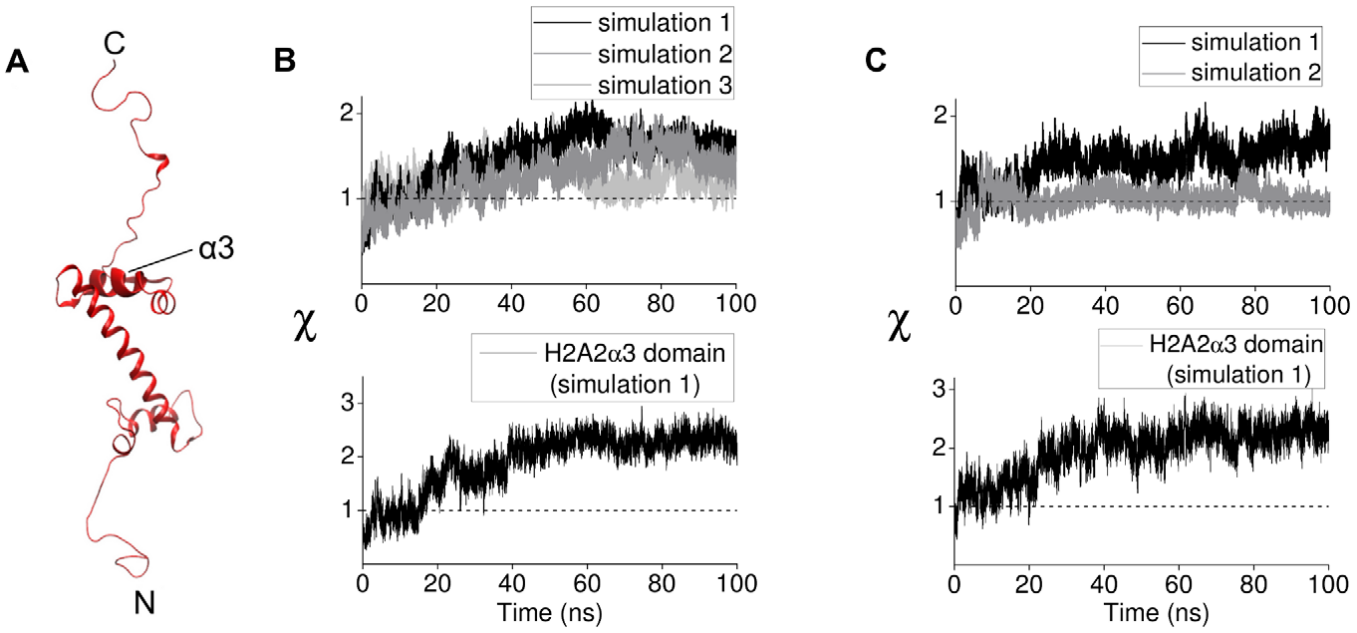
Structural changes in histone H2A(2) during tail-truncated simulations. (A) Structure of histone H2A with position of the 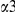 domain shown by thickened helix. (B) *Upper panel*. Order parameter 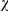 for H2A(2) fold domain from three independent H3 tail-truncated nucleosome simulations. *Lower panel*. Order parameter 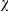 for H2A(2)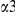 domain from the H3 tail-truncated nucleosome simulation 1. (C) *Upper panel*. Order parameter 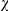 for H2A(2) fold domain from two independent H2A tail-truncated nucleosome simulations. *Lower panel*. Order parameter 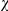 for H2A(2)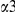 domain from the H2A tail-truncated nucleosome simulation 1.

To illustrate how tail truncation affects nucleosome structure 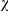 is plotted for each copy of the four histones over the 100 ns trajectories obtained from the four tail-truncated nucleosome simulations (Fig. 4B). Tail truncation does not affect the stability of most of the histone monomers. However, when the H3 or H2A tails are truncated, one of the two copies of histone H2A exhibits a persistently increased RMSD relative to the other cases (see the box in Fig. 4B). We call this monomer H2A(2) from now on. This indicates that H3 or H2A tail truncation destabilizes the nucleosome structure. Two further independent simulations of the H3 tail-truncated nucleosome confirmed the structural changes in H2A(2) upon H3 tail removal (Fig. 5B *upper panel*).

To locate the structural domain of the H2A monomer that is responsible for the increased RMSD, we computed 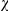 for each structural domain of H2A(2) from the H3 tail-truncated simulation. The 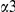 domain of H2A was found to exhibit an increase in the value of 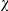 similar to that observed for the entire H2A monomer from the same trajectory (Fig. 5B). The RMSD of the same domain also increases when the H2A tail is truncated (Fig. 5C).

The simulations reported here are non-equilibrium simulations each of which can follow a different pathway. However, we found that certain changes involving the H2A(2)
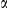
3 domain are common to all the simulations which gives statistical significance to our result. The differences in order parameters among replicate simulations in Fig. 5B and 5C are commented in the Discussion.

**Figure 6:**
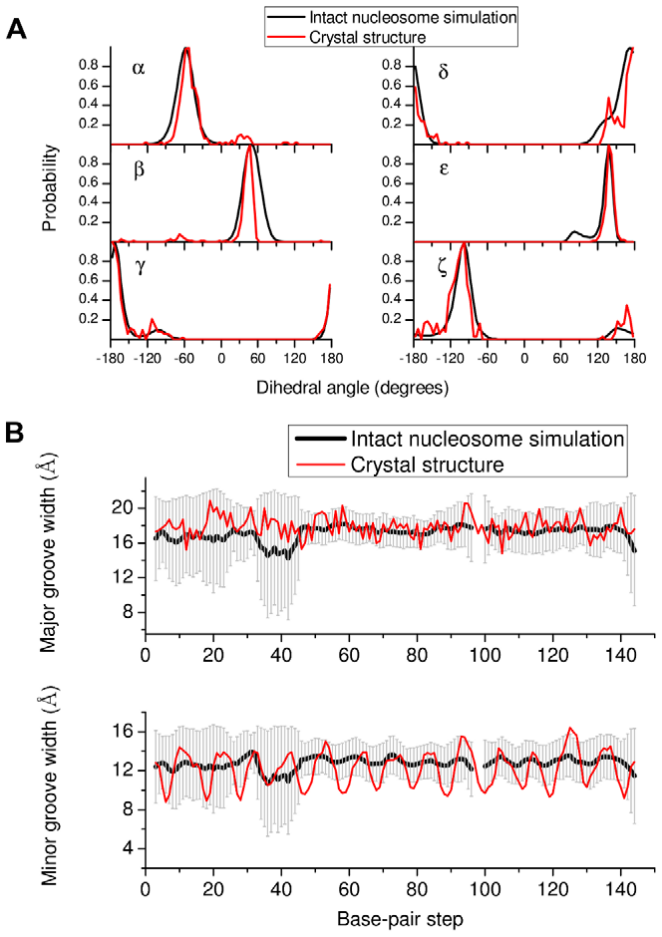
NA backbone and groove characteristics. (A) Probability distributions for DNA backbone dihedral angles. The dihedral angles 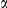
(O3'-P-O5'-C5'), 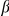
(P-O5'-C5'-C4'), 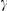
(O5'-C5'-C4'-C3'), 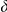
(C5'-C4'-C3'-O3'), 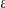
(C4'-C3'-O3'-P) and 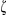
(C3'-O3'-P-O5') obtained from the intact nucleosome simulation are compared with those from the crystal structure (1KX5.pdb). (B) DNA major and minor groove width fluctuations along the sequence (chain I) in the intact nucleosome simulation. Groove widths are calculated as P-P distances using the algorithm of Hassan and Calladine [46] implemented in 3*DNA* [30].

Realistic parametrization of force fields for the nucleic acids has been a long-standing problem [31]–[33] and CHARMM22 force field is known to overstabilize A form of DNA [34]. Major shortcomings of the CHARMM22 force field have been overcome in CHARMM27 [31], [32] which is used here. In Fig. 6 we plot the probability distribution for the phosphodiester backbone dihedrals from the intact nucleosome simulation and compare them with the distributions obtained from the crystal structure. The relative smoothness of the simulation distributions originates from the dynamics of the system that is not taken into account by the crystallographic average structure. Most of the structures show BI type (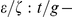) conformation. Furthermore, the 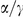 dihedral states present in the crystal structure also persist during the simulation. It is of interest to note that the above parameters, calculated using the CHARMM27 force field are in agreement with those obtained using the AMBER force field on the same molecule [19]. The DNA helical parameter fluctuations are also in good agreement with those extracted from the crystal structure (Supplementary Fig. S2).

While analyzing the DNA dihedral parameters we did find that one nucleotide base (Cyt49(J)) is unstacked and the neighboring bases (Gua98(I)-Cyt50(J)) show unusual (non Watson-Crick) base-pairing in the intact nucleosome simulation. However, this did not lead to any unusual fluctuation in the neighboring amino-acid residues. Furthermore, since the DNA backbone and helical parameter values derived from all other base pairs are in agreement with x-ray and simulation data, this may not be artefactual.

DNA major and minor groove characteristics are plotted in Fig. 6B and are again in agreement with the crystal structure. The minor grooves exhibit a periodic variation of width with the minima corresponding to the base-pair steps contacting histone arginines that is of smaller amplitude than the crystal structure, again possibly due to the dynamics. A larger fluctuation in DNA groove width is observed for the base pair steps 1–40 (chain I) with the largest fluctuation near base-pair step 40 being caused by Arg11 of histone H2A(1) probing the DNA binding sites.

**Figure 7:**
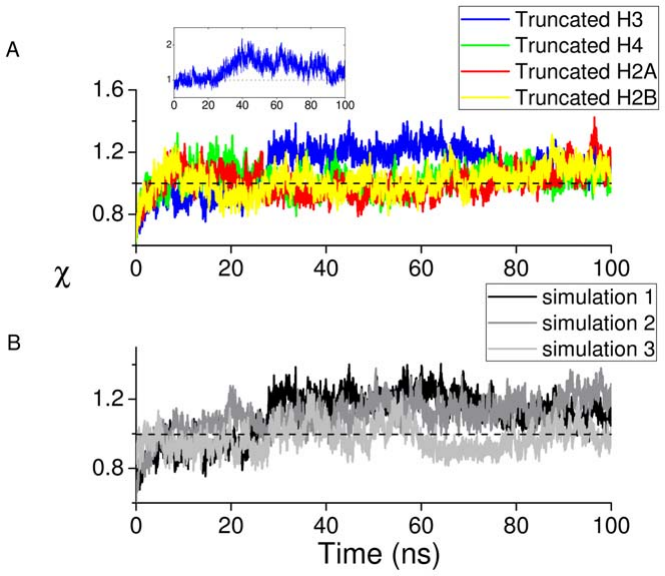
Destabilization of DNA upon tail truncation. (A) Order parameter 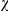 for DNA from the four tail-truncated nucleosome simulations. The inset shows the order parameter for the DNA segment between the dyad and SHL +1.5 for the H3 tail-truncated nucleosome simulation. (B) The order parameter 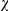 for DNA from three independent H3-tail truncated nucleosome simulations.

In the intact nucleosome simulation the DNA RMSD stabilizes around 3.2 Å (Supplementary Fig. S3), which is a relatively small value for a system of large radius of gyration (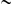
50 Å). The effect of tail truncation on the nucleosomal DNA was also examined and it was found that only the H3 tail truncation affects DNA stability (Fig. 7A). Further, independent simulations also confirmed DNA destabilization upon H3 tail truncation (Fig. 7B). To determine to which segment of the DNA the increased RMSD corresponds, the nucleosomal DNA was divided into 14 segments based on the SHLs shown in Fig. 1. The results indicate that H3 tail truncation destabilizes the segment of DNA between the dyad and SHL +1.5 (Fig. 7A inset).

### Analysis of Nucleosomal Interactions

We analyzed the alteration of residue interactions upon tail truncation for the H2A(2) 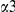 domain and the H2A C terminal domain for the following reasons - i) the order parameter plots showed destabilization of the H2A(2) 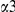 domain and, ii) it has been suggested that the H2A C terminus contacts with surrounding residues may play a key role in nucleosome stability [20].

#### Residues in the H2A(2) 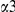 domain

**Figure 8:**
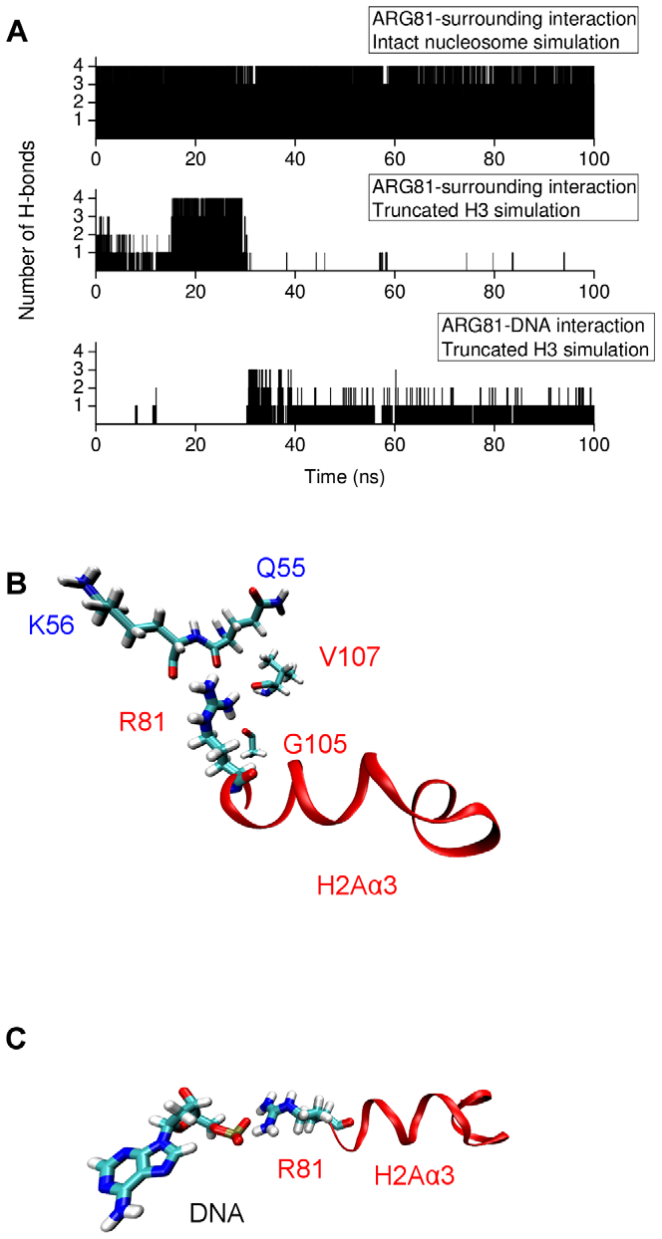
Interactions with Arg81 of H2A(2) during the intact and H3 tail-truncated simulations. (A) Number of H-bonds between Arg81 and selected surrounding residues from intact and H3 tail-truncated simulations. The selected surrounding residues are shown in (B) and (C). (B) Arg81 H-bonds with Gln55 and Lys56 of H3(1) and Gly105 and Val107 of H2A(2) during the intact nucleosome simulation. (C) Arg81 H-bonds with DNA in the H3 tail-truncated simulation.

Analysis of the trajectories with truncated H3 tails revealed an active role of histone arginines in structural alterations in the H2A(2)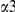 domain. Arginines are highly flexible positively-charged residues with long side chains. The crystal structure of the nucleosome core particle shows an intricate network of arginine-DNA interactions in which the arginines are found mainly in the DNA minor grooves [2]. In the intact nucleosome simulation Arg81 of H2A(2) is stably hydrogen bonded with Gln55 and Lys56 of H3(1) and Gly105 and Val107 of H2A(2). In the absence of the H3 tails, the hydrogen bonds between Arg81 and surrounding residues were broken and new hydrogen bonds were formed between Arg81 and the nucleosomal DNA (Fig. 8 and Supporting video S1,S2).

**Figure 9:**
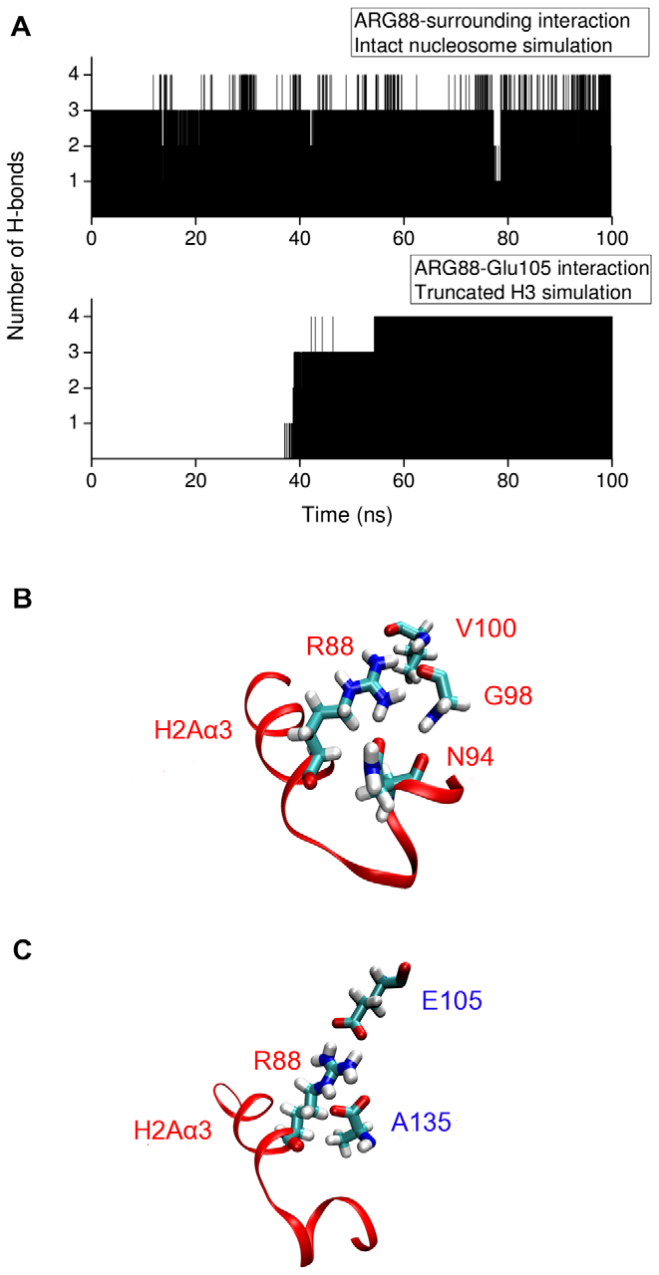
Interactions with Arg88 of H2A(2) during the intact and H3 tail-truncated simulations. (A) Number of H-bonds between Arg88 and selected surrounding residues from intact and H3 tail-truncated simulations. The selected surrounding residues are shown in (B) and (C). (B) Arg88 H-bonds to Asn94, Gly98 and Val100 of H2A(2) during the intact nucleosome simulation. (C) Arg88 H-bonds to Glu105 of H3(1) and Ala135 of H3(2) in the H3 tail-truncated simulation.

Concomitant with the above Arg81 interaction change, there is also a change of interaction of the nearby Arg88 of H2A(2) (Fig. 9). Whereas in the intact nucleosome simulation Arg88 is hydrogen bonded to Asn94, Gly98 and Val100 of H2A(2), in the truncated H3 simulation this hydrogen bond network is broken and Arg88 makes stable hydrogen bonds with Glu105 of H3(1) and Ala135 of H3(2) (Fig. 9 and Supporting video S3,S4).

**Figure 10:**
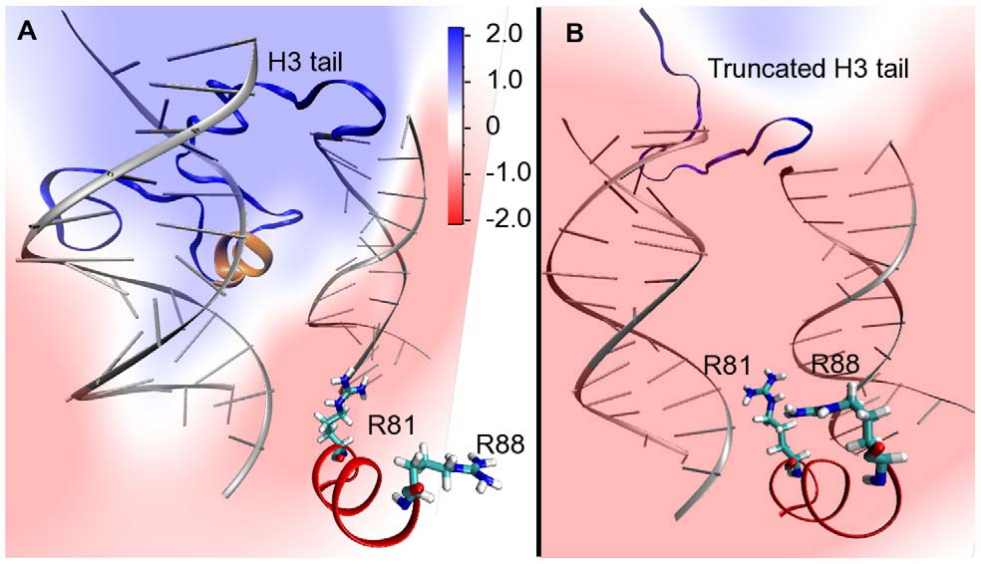
Electrostatic potential maps of DNA and H3 N-terminal tail as seen by Arg81 and Arg88. Potential is color coded (in units of Volts) as shown in the scale. (A) Snapshot from the intact nucleosome trajectory showing that in presence of the H3 N-terminal tail Arg81 and Arg88 points away from the DNA surface. The location of the 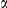-helix in H3 tail is shown in orange ribbons. (B) Snapshot from the H3 tail truncated simulation showing the Arg81 and Arg88 sidechain pointing towards the DNA in the absence of the H3 tail.

The change of interaction of arginines upon truncation of the H3 tails is likely a result of local change of electrostatic environment (Fig. 10). In the intact nucleosome simulation the DNA primarily interacts with basic amino acids from the 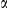-helical domain of the H3 tail and the arginines see a positive potential. On removal of H3 tail the DNA loses interactions with the basic residues from the 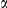-helix and become available for interaction with arginines.

Interestingly, truncation of the H2A tails (simulation 1) causes interaction changes in the same H2A(2)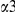 domain as does truncation of the H3 tails. A detailed analysis of the truncated H2A simulation revealed that this occurs due to breaking of hydrogen bonds of Arg88 with the surrounding residues. In a very similar manner to when the H3 tails were truncated, Arg88 formed new hydrogen bonds with Glu105 of H3(1) and Ala135 of H3(2). In the second independent simulation with truncated H2A tails (simulation 2) Arg88 formed hydrogen bonds only with Ala135 of H3(2).

#### Residues in the H2A C terminal domain

**Figure 11:**
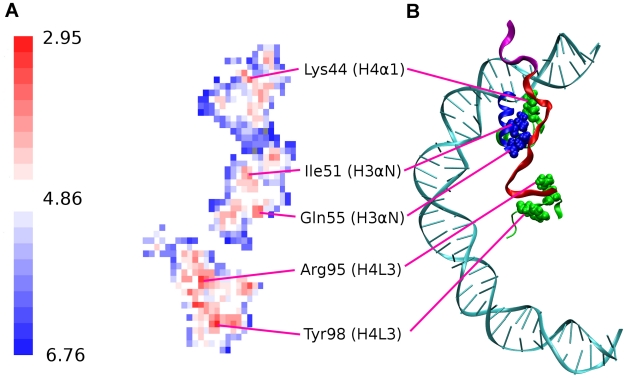
Interaction of H2A C terminal extension (amino acids 100–129) with surrounding residues. (A) *Molsurfer* [47] generated contact map of H2A C terminal docking domain (amino acids 100–119) interaction surface as derived from interatomic distances calculated from the structure averaged over the 100 ns of the intact nucleosome trajectory. Distances (in units of Å) are color coded as shown in the scale. Residues in close contact with H2A C terminus are indicated. (B) Positions of the H2A C terminal docking domain (colored red) close contact residues are shown in the nucleosome structure. The end of the H2A C terminus (amino acids 120–129) is colored magenta.

The crystal structure of the nucleosome core particle shows that the histone H2A C terminus lies close to the H3 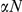, H3 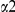, H4 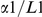, and H4 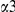 domains [2]. To examine which residues from these domains are in contact with the H2A C terminus, a contact map of the C terminus with the surrounding structural elements was calculated for the structure averaged over 100 ns of intact nucleosome simulation (Fig. 11A). The contact map is a two dimensional projection of the three dimensional interface between the C terminus and surrounding residues (see Supplementary Fig. S5 and Text S2). A distance value is assigned to each interface point which is sum of the distances to the closest atoms from the residues forming the interface. The contact map reveals that the residues in close contact with the H2A C terminus comprise Lys44 (H4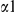), Ile51 (H3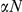), Gln55 (H3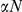), Arg95 (H4L3) and Tyr98 (H4L3) of which Lys44, Ile51 and Gln55 are close to the C terminal-DNA contact region.

**Figure 12:**
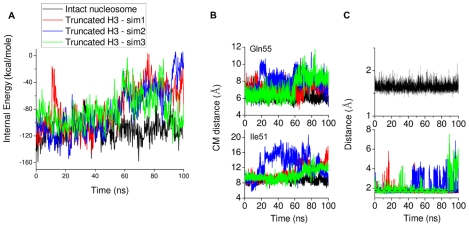
Destabilization of H2A docking domain interaction with Ile51, Gln55 and the DNA upon H3 tail truncation. (A) Interaction energy (vdw+electrostatics) between H2A docking domain and surrounding amino acids (B) Time series of the distance between the centers of mass of Ile51 or Gln55 and the nearest residue on the H2A C terminus (Leu115 and Asn110, respectively) is plotted. (C) Destabilization of H2A C terminus interaction with DNA upon H3 tail truncation. Time series of the minimum distance between Lys118 and Lys119 of H2A C terminus and the DNA is plotted. An increase of the distance between the C terminus end (Lys118 and Lys119) and DNA was considered to be a detachment if the minimum atomic distance between them during a tail-truncated simulation was greater than the minimum distance averaged over the intact nucleosome trajectory plus its standard deviation. The black line (dotted) indicates the minimum distance between C terminus and the DNA (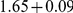 Å) below which the C terminus is ‘in contact’ with the DNA.

Truncation of the H3 tail destabilizes contact of the H2A docking domain with the surrounding amino acids (Fig. 12A). In Table 1 we show results from experimental tail-truncation and alanine mutagenesis studies on the nucleosome [12]. Interestingly, mutation of Ile51 or Gln55, which are in close contact with H2A C terminus, markedly increases both the nucleosome sliding rate and histone dimer exchange. It is likely that these mutations destabilize the contact of the C terminus with Ile51 and Gln55. Since the effects on nucleosome sliding and dimer exchange observed upon H3 tail truncation and Ile51 or Gln55 mutation are similar, H3 tail truncation may also involve destabilization of the interaction between the C terminal contact and Ile51 and/or Gln55. In Fig. 12B are plotted the distances between the centers of mass of Ile51 and Gln55 and the residues in close contact with them on the H2A C terminus for the simulations with truncated H3 tails. The plots indicate that Ile51 and Gln55 form stable contacts during the intact nucleosome simulation (black line) but undergo large positional fluctuations upon H3 tail truncation.

**Table 1:** Experimental results on the effect of alanine mutagenesis and tail-truncation on nucleosome dynamics.

Sequence modification	Octamer formation	Nucleosome sliding rate relative to WT^a^	Histone dimer exchange relative to WT
Ile51 mutation	−	9.2 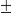 1.0	3.4 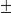 0.7
Gln55 mutation	−	5.4 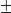 0.4	3.4 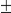 0.5
H3 tail truncation ( 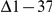 )	+	Nucleosome unstable	3.1 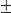 0.3
H2A tail truncation ( 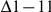 , 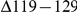 )	x^b^	2.4 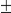 0.2	x

Experimental data is obtained from Ref. [12].

a ‘WT’ = wild type.

b ‘x’ = data not available.

Destabilization of the H2A C terminus upon H3 tail truncation affects the C terminus-DNA contact. In the intact nucleosome simulation the H2A C terminus (Lys118 and Lys119) makes stable contact with the DNA through hydrogen bonding. Upon H3 tail truncation the C terminus switches between states ‘in contact’ with DNA and ‘detached’ (Fig. 12C).

**Figure 13:**
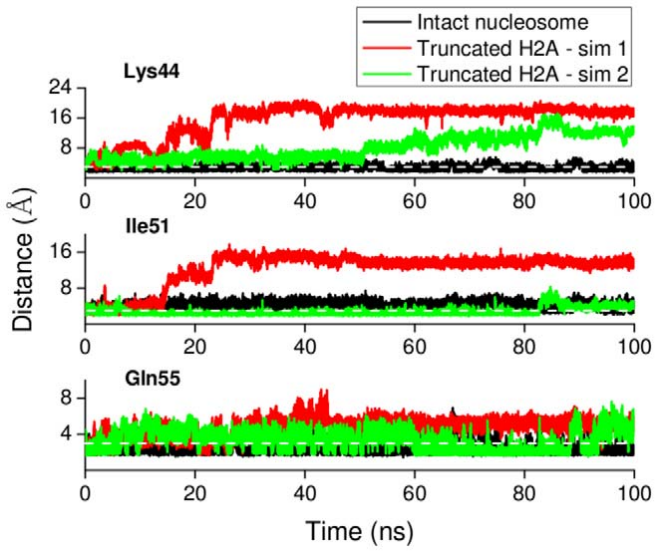
Interaction of close contact residues from **Fig. 11**, located near the region where H2A C terminus contacts the dyad, for intact and truncated H2A simulations. The minimum distances between any atom of Lys44, Ile51 or Gln55 and the closest residue on the H2A C terminus (Leu116, Leu115 and Asn110, respectively) are plotted as a function of time. The white line (dotted) indicates the distance (3 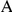) below which residues can be regarded to be in close contact.

Truncation of H2A tails removes H2A C terminus-DNA contacts. This affects the remaining part of the H2A tail differently in the independent simulations : in simulation 1 the H2A docking domain breaks contact with Lys44 and Ile51, in the other only minimal loss of contact with Lys44 is observed (Fig. 13). Further comments on this are added in paragraph 6 of the Discussion.

## Discussion

The aim of the present study was to provide atomic-level information on interactions within the nucleosome that are altered upon tail truncation. This was accomplished by multiple all-atom MD simulations of intact and tail-truncated nucleosomes, with each trajectory covering a time frame 5 times longer than previously-reported simulations comparing intact and tail-truncated nucleosomes [19]. Several independent simulations were performed, totalling 800 ns of combined trajectory. A comparison of the key results obtained from the 20 ns [19] and 100 ns trajectories are given in the Supplementary Table S1.

The formation of 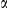-helical structure in one of the H3 tails may be a result of specific histone-DNA interactions (Fig. 3B). The starting structure for our simulations is the 1.9 Å crystal structure of the nucleosome core particle [2] in which residues from the two copies of the same type of histone tail domains have been assigned different order values. This affects the structural changes in the nucleosome during the simulation and it is likely that the asymmetric structural changes in histone H3 occur due to the chosen starting structure. Recent studies indicate that amino acid residues in this 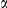-helical domain are crucial post-translational modification sites, e.g., Lys14 is an essential p300 acetylation substrate required for dissociation of the histone octamer from the promoter DNA [35], and methylation at Arg17 is linked to gene activation [36].

Hyperacetylation of lysine residues neutralizes its positive charge by transferring an acetyl moiety onto the 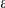-amino group which reduces the lysine-DNA interactions. In the simulations similar change of electrostatic environment may arise from removal of lysine residues. This is indirectly realized in our H3 tail-truncated simulations as mentioned below: in the intact nucleosome simulation we observed 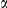-helix formation by residues 14–20 of H3 tail. Interestingly, Lys14 and Lys18 of this alpha-helix domain belong to the known acetylation sites of histone [37]. Thus, the removal of H3 tail (residues 1–26) disrupts interactions between these lysines and the DNA in a way similar to acetylation of lysines and likely induces change of interaction in argines through the electrostatic mechanism described here (Fig. 10).

The reason for observing interaction changes for one H2A monomer in H3 tail-truncated simulations is not clearly understood (a comparison of interaction changes between histone monomers is provided in Supplementary Table S2). We note that the structural changes were observed in the H2A monomer lying close to the H3 tail which showed a propensity for 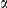-helix formation during the intact nucleosome simulation. Thus, disruption of H3 tail 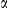-helix-DNA interaction is likely correlated with the structural changes in H2A. Alternatively, the effect may also be due to incomplete conformational sampling and similar structural changes would be observed in the other H2A monomer on longer time scale. On truncation of H2A tail, a similar change of interactions in the H2A docking domain is observed for both the H2A monomers in the molecule. However, this case is different from H3 tail-truncated simulations, because the effects observed here are likely due to truncation of H2A tails, whereas in other case it is likely due to disruption of 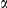-helix-DNA interactions in the simulation.

**Figure 14:**
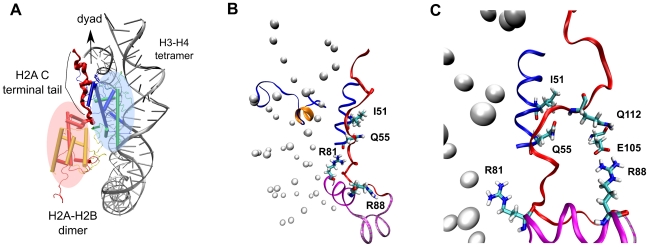
Regulation of nucleosome stability through H2A docking domain contacts. (A) Positions of the H2A–H2B dimer and H3–H4 tetramer in a nucleosome illustrating that the interaction surface between the H2A–H2B dimer and H3–H4 tetramer is provided by the H2A C terminal tail. Histone dimers H3, H4, H2A and H2B are colored in blue, green, red and yellow, respectively. Part of the nucleosome structure is shown in cartoon representation for clarity of vision. (B)–(C) Disruption of contacts between the H3 tail 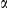-helix (colored orange) and the DNA triggers change of interaction of histone arginines (Arg81 and Arg88). Newly formed polar contacts between Arg88, Glu105 and Gln112 destabilizes interaction of the H2A docking domain with closely lying amino acids (Ile51 and Gln55). Histone protein domains are shown as ribbons and DNA phosphorous atoms are shown as spheres. Histone domains are color coded as follows : H3
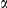
N (blue), H2A
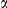
3 (magenta), H2A
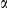
C (grey), H2A docking domain (red).

The H2A docking domain provides the interaction surface between the histone H3–H4 tetramer and the H2A–H2B dimer (Fig. 14A). Destabilization of the H2A docking domain is likely to weaken the dimer-tetramer interaction and affect nucleosome stability. Recent work has shown that the C-terminally truncated nucleosome is less stable and has higher mobility than the wild type H2A-containing nucleosome [20], [38], [39]. Since stability of the docking domain depends largely on the amino acids in close contact with this region, any disruption of the interactions with these amino acids would alter nucleosome stability. In the present simulations weakening of docking domain contact interactions (Fig. 12) gives a possible explanation for increased histone dimer exchange rate upon H3 tail truncation [12]. Furthermore, the simulations provide insight into two possible mechanisms of destabilization of the docking domain (Fig. 14 B–C). The direct mechanism involves altering the residues in contact with the docking domain, exemplified by alanine mutagenesis *in vitro* studies [12]. In a cellular environment direct alteration of H2A docking domain contacts might also be achieved by incorporation of histone variants, such as H2ABbd or H2A.Z, which differ in amino acid sequence from the canonical H2A. The alternative mechanism involves breaking of specific contacts between the H3 tail and the DNA, thus triggering destabilization of the H2A docking domain through changes of interaction of the arginines in the H2A 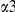 domain.

In the simulations we observe a correlation between the breaking of contacts of the H2A docking domain with close by amino acid residues and the change of interaction of Arg88 of the H2A(2)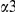 domain. Hydrogen bond formation between Arg88(H2A) and Glu105(H3) is simultaneous with the breaking of contacts of Ile51 and Gln55 with the H2A docking domain. It is likely that the change of interaction of Arg88 also changes the electrostatic environment in the vicinity of the H2A docking domain and new polar contacts between Arg88(H2A), Glu105(H3) and Gln112(H2A) are formed (Fig. 14C). When the Arg88-Glu105 contact is not formed in the simulations (H2A tail-truncated simulation 2) minimal loss of contact of Ile51 and Gln55 with the H2A docking domain is observed (Fig. 13).

We also found that certain changes involving the alteration of sidechain hydrogen bonding of Arg88 are common to all the H3 and H2A tail-truncated simulations (see Supplementary Fig. S4). Replicate simulations in Fig. 5B and 5C follow different pathways between the beginning (when Arg88 is hydrogen bonded to Asn94, Gly98 and Val100) and the end (when Arg88 is hydrogen bonded to Glu105 and Ala135) states which results in the differences in the order parameters in the plots. In Fig. 5C upper panel, the data seem different among replicates since the end state is different (Arg88 is hydrogen bonded to Ala135 only) in this case.

It has been proposed that transient opening of outer turns of DNA facilitate nucleosome sliding by capturing loops on the nucleosome surface [40], [41] and experimental work has reported that the DNA ends open up upon H3 tail deletion [12], [42]. However, we do not observe opening of DNA ends in the present simulations. This is likely to arise from the fact that whereas in the experiments of Ref. [12] the nucleosomes were assembled onto 181 bp of DNA fragments derived from 601.3 strong nucleosome positioning sequence [43], the structure simulated here consists of only 147 bp of nucleosomal DNA. Hence the present structure does not contain DNA ends extending into the solvent.

In the simulations a segment of DNA between the dyad and SHL +1.5 is destabilized as a result of H3 tail truncation (Fig. 6A inset). Truncation of the H3 tail (amino acids 1–26) removes the hydrogen bonds between the tail residues and the DNA found in the crystal structure. Charged residues from remaining part of the H3 tail (amino acids 27–45) then probe between possible DNA binding sites in the simulations which is observed as an increase the value of the order parameter for this part of the DNA.

It has been proposed that histone core domain modifications may alter nucleosome mobility [7]. The allosteric change of interactions in the H2A 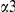 domain and C terminal tail upon H3 and H2A tail truncation probed here may reveal potential ways in which dynamical properties of the nucleosome can be manipulated. Truncation of the C terminal tail affects the binding of ATP-dependent chromatin remodelling factors [20], further suggesting an important role of this domain in nucleosome mobility. The H2A C terminus has also been found to be crucial for binding of the linker histone H1 to nucleosome [20]. Core domain modification, by providing marks for recruitment of chromatin binding proteins, have the potential to play a vital role in gene regulation.

## Supporting Information

Text S1 **B-factor for nucleosomal DNA.** DNA phosphorous atom B-factors were computed from the last 50 ns of the intact and tail-truncated nucleosome simulations (Fig. S1) [48], [49]. Small B-factor differences between intact and tail-truncated nucleosome simulation are observed at specific nucleotide positions where the truncated histone tails contact the DNA in the intact nucleosome.
(PDF)

Text S2 **Finding H2A docking domain contacts.** Through the contact map analysis we want to find contact residues between the H2A docking domain and its surrounding which also form an interacting pair. To do this it is necessary to verify the contact map based information with visualization in 3D. This is achieved by the *Molsurfer* program which, in addition to the 2D map, has an interface for viewing in 3D (*WebMol*) as shown in Fig. S19. In the Figure the 2D contact map is shown the left panel whereas in the right panel the docking domain and its surrounding are shown in 3D in backbone representation. In the program when the cursor is pointed to a grid location on the 2D map the corresponding position is shown on the 3D interface by a red dot. One can then ‘focus’ (or zoom in) on this red dot to see which residues are forming an interacting pair and are in contact. Furthermore, the residue contacts found using *Molsurfer* are validated by visualizing the trajectory in *VMD*.
(PDF)

Figure S1 **DNA phosphorous atom B-factors obtained from X-ray crystallography (dotted line) and those computed from the last 50 ns of intact and tail-truncated nucleosome simulations (continuous lines).** The B-factors are shown for the two chains of DNA: I and J. The labels under the curves indicate the histone chains and secondary structure elements that make intermolecular contacts with the DNA.
(PDF)

Figure S2 **DNA helical parameter fluctuations during intact nucleosome simulation.** Average helical parameters with fluctuations (standard deviation) indicated as error bars are compared with those obtained from the crystal structure (1KX5.pdb).
(PDF)

Figure S3 **DNA phosphorous atom RMSD versus simulation time for intact nucleosome.**
(PDF)

Figure S4 **Number of hydrogen bonds between Arg88 of H2A(2) and Glu105 of H3(1) or Ala135 of H3(2) as a function of time for tail-truncated nucleosome simulations.** In the H2A tail-truncated nucleosome simulation number 2 no hydrogen bond was formed between Arg88 and Glu105.
(PDF)

Figure S5 **The 2D contact map of the H2A docking domain with the** ***WebMol*** **interface for viewing the docking domain and its surrounding in 3D.** In the *WebMol* interface atoms are shown in backbone representation. The interface between the H2A docking domain and its surrounding appears as a mesh.
(PDF)

Table S1 **Comparison between 20 ns** [**19**] **and 100 ns nucleosome trajectories.**
(PDF)

Table S2 **Interaction change in histone monomers with respect to key findings.**
(PDF)

Video S1 **Arg81 interacting with Gln55 and Lys56 of H3(1) and Val107 of H2A(2) in the intact nuclesome simulation.**
(MPG)

Video S2 **Time course of interaction changes in Arg81 during the H3 tail-truncated simulation.**
(MPG)

Video S3 **Arg88 making stable hydrogen bonds to Asn94, Gly98 and Val100 of H2A(2) in the intact nucleosome simulation.**
(MPG)

Video S4 **In the H3 tail-truncated simulation Arg88 sidechain moves towards the DNA and then makes stable hydrogen bonds to Glu105 of H3(1) and Ala135 of H3(2).**
(MPG)
