# Supplementary material for: Role of Histone Tails in Structural Stability of the Nucleosome
Source: PLoS Comput Biol. 2011 Dec 15;7(12):e1002279. doi: 10.1371/journal.pcbi.1002279 (PMC3240580; doi:10.1371/journal.pcbi.1002279)
Supplement: Text S1 — B-factor for nucleosomal DNA. DNA phosphorous atom B-factors were computed from the last 50 ns of the intact and tail-truncated nucleosome simulations (Fig. S1) [48], [49]. Small B-factor differences between intact and tail-truncated nucleosome simulation are observed at specific nucleotide positions where the truncated histone tails contact the DNA in the intact nucleosome. (PDF) [file pcbi.1002279.s001.pdf]

DNA phosphorous atom B-factors were computed from the last 50 ns of the intact and tail-truncated nucleosome simulations (Fig. S1) [48,49]. Small B-factor differences between intact and tail-truncated nucleosome simulation are observed at specific nucleotide positions where the truncated histone tails contact the DNA in the intact nucleosome.
