# Supplementary material for: Role of Histone Tails in Structural Stability of the Nucleosome
Source: PLoS Comput Biol. 2011 Dec 15;7(12):e1002279. doi: 10.1371/journal.pcbi.1002279 (PMC3240580; doi:10.1371/journal.pcbi.1002279)
Supplement: Text S2 — Finding H2A docking domain contacts. Through the contact map analysis we want to find contact residues between the H2A docking domain and its surrounding which also form an interacting pair. To do this it is necessary to verify the contact map based information with visualization in 3D. This is achieved by the Molsurfer program which, in addition to the 2D map, has an interface for viewing in 3D (WebMol) as shown in Fig. S19. In the Figure the 2D contact map is shown the left panel whereas in the right panel the docking domain and its surrounding are shown in 3D in backbone representation. In the program when the cursor is pointed to a grid location on the 2D map the corresponding position is shown on the 3D interface by a red dot. One can then ‘focus’ (or zoom in) on this red dot to see which residues are forming an interacting pair and are in contact. Furthermore, the residue contacts found using Molsurfer are validated by visualizing the trajectory in VMD. (PDF) [file pcbi.1002279.s002.pdf]

Through the contact map analysis we want to find contact residues between the H2A docking domain and its surrounding which also form an interacting pair. To do this it is necessary to verify the contact map based information with visualization in 3D. This is achieved by the *Molsurfer* program which, in addition to the 2D map, has an interface for viewing in 3D (*WebMol*) as shown in Fig. S5. In the Figure the 2D contact map of the H2A docking domain with its surrounding is shown the left panel whereas in the right panel the docking domain and its surrounding is shown in 3D in backbone representation. In the program when the cursor is pointed to a grid location on the 2D map the corresponding position is shown on the 3D interface by a red dot. One can then 'focus' (or zoom in) on this red dot to see which residues are forming an interacting pair and are in contact. Furthermore, the residue contacts found using *Molsurfer* are validated by visualizing the trajectory in *VMD*.
