# Supplementary figures and images for: Role of Histone Tails in Structural Stability of the Nucleosome
Source: PLoS Comput Biol. 2011 Dec 15;7(12):e1002279. doi: 10.1371/journal.pcbi.1002279 (PMC3240580; doi:10.1371/journal.pcbi.1002279)

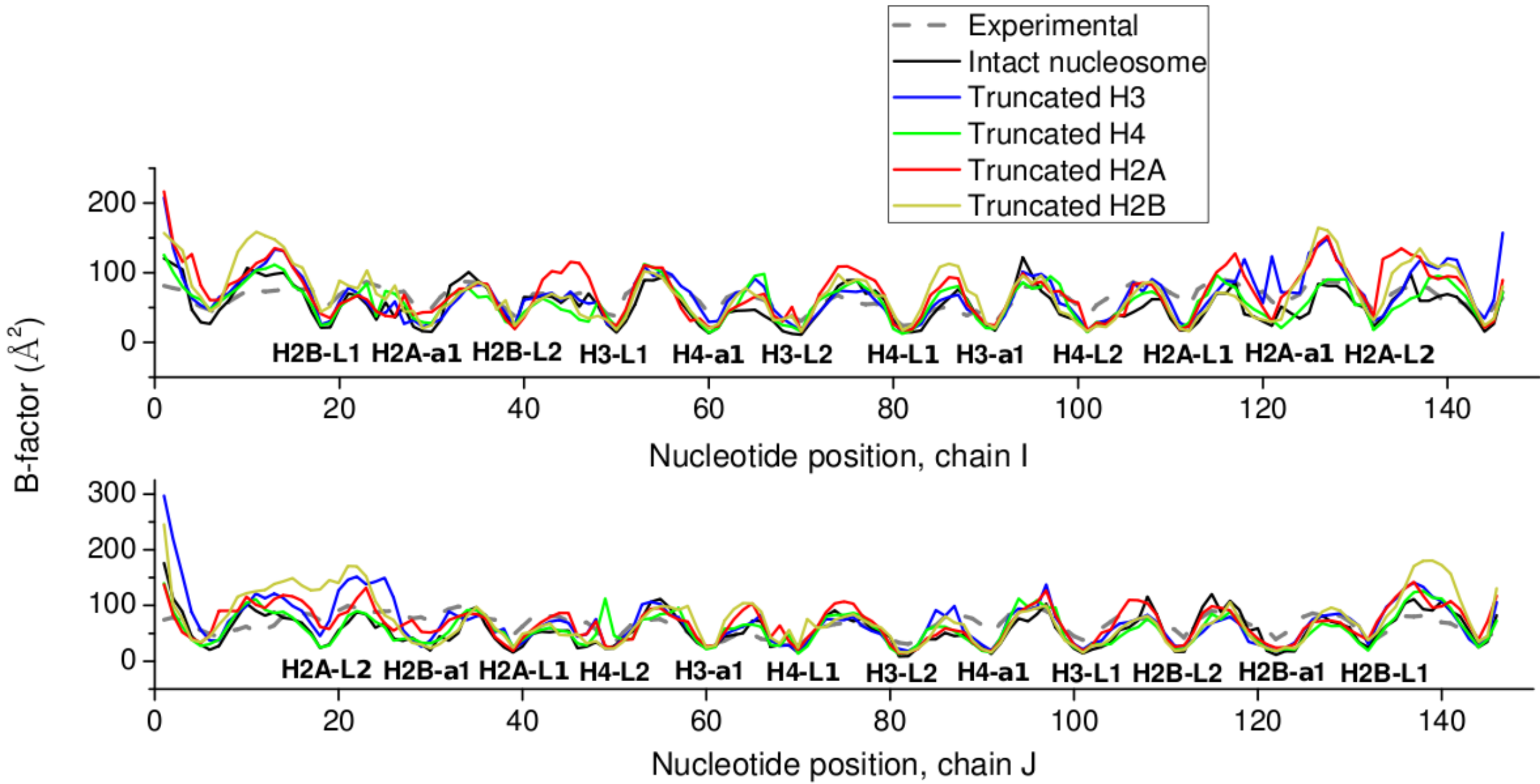

Supplement: Figure S1 — DNA phosphorous atom B-factors obtained from X-ray crystallography (dotted line) and those computed from the last 50 ns of intact and tail-truncated nucleosome simulations (continuous lines). The B-factors are shown for the two chains of DNA: I and J. The labels under the curves indicate the histone chains and secondary structure elements that make intermolecular contacts with the DNA. (PDF) [file pcbi.1002279.s003.pdf]

— Intact nucleosome simulation  
— Crystal structure

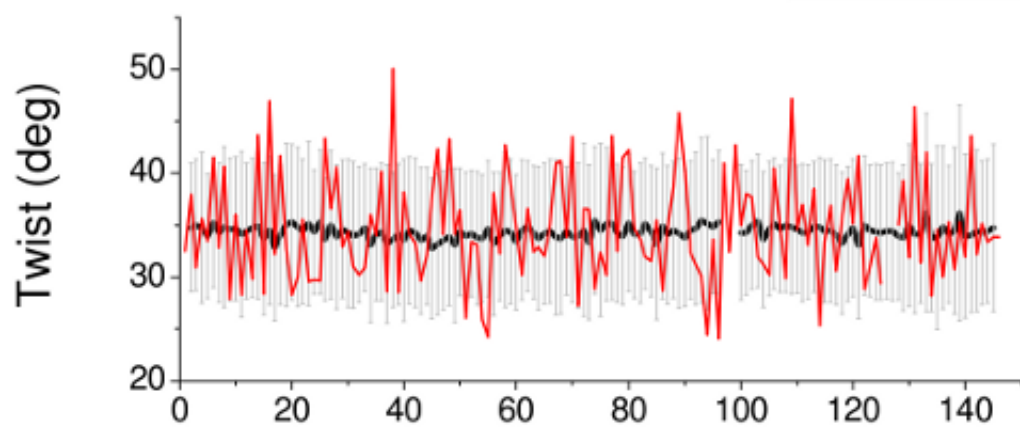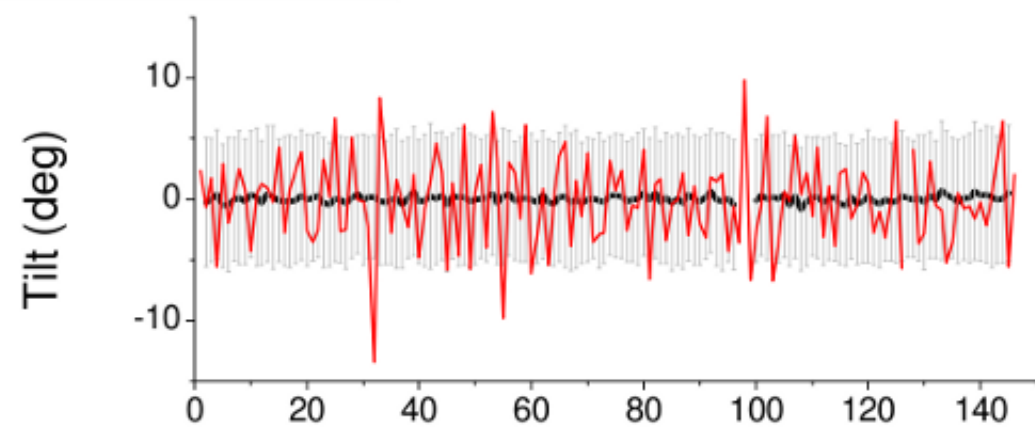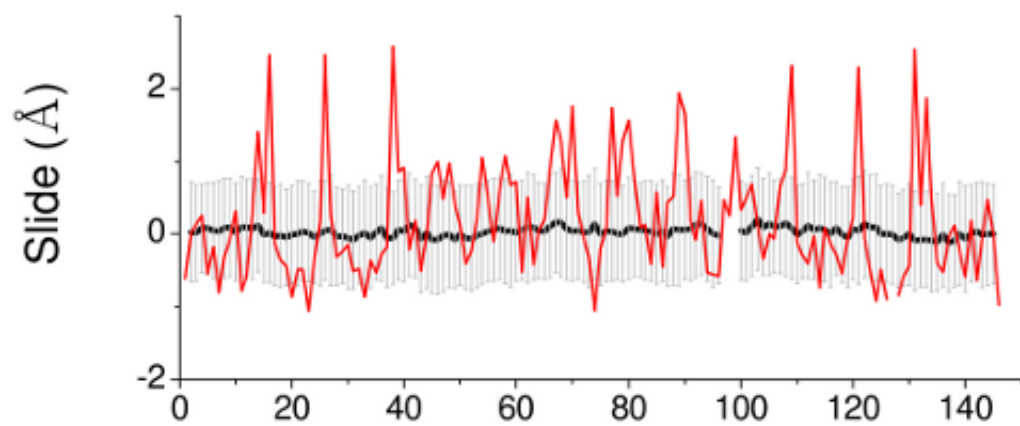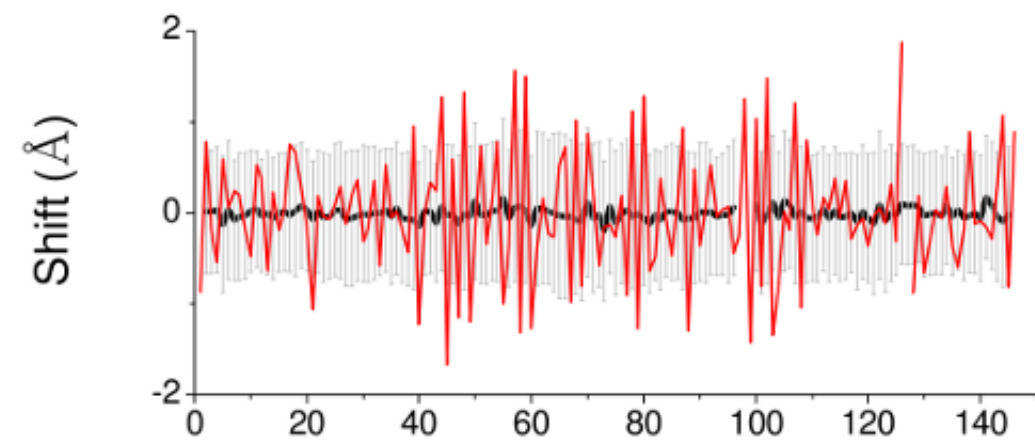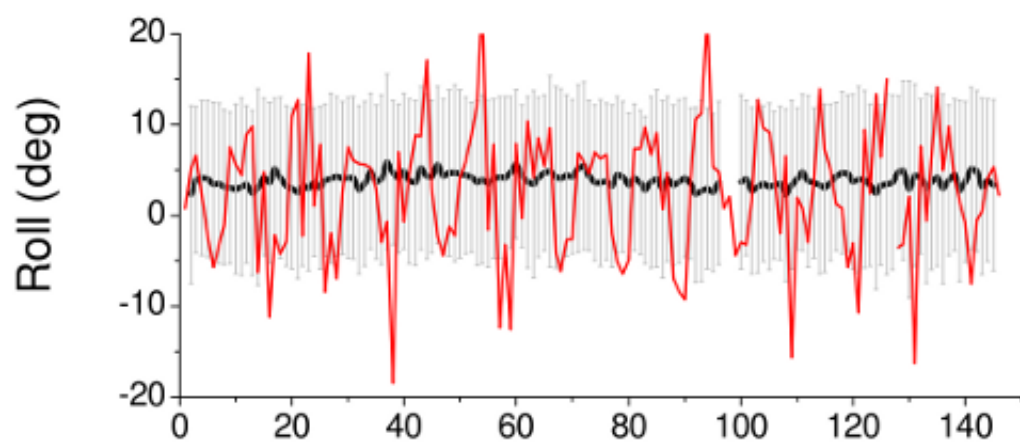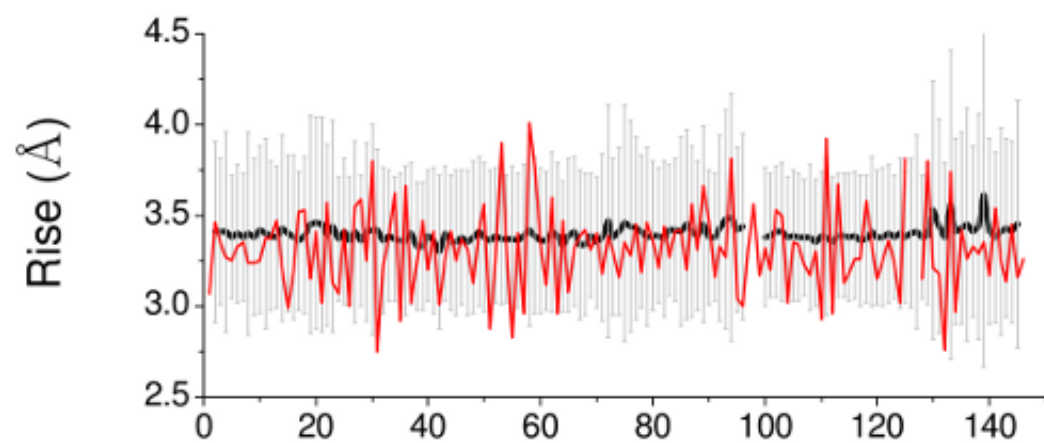

Supplement: Figure S2 — DNA helical parameter fluctuations during intact nucleosome simulation. Average helical parameters with fluctuations (standard deviation) indicated as error bars are compared with those obtained from the crystal structure (1KX5.pdb). (PDF) [file pcbi.1002279.s004.pdf]

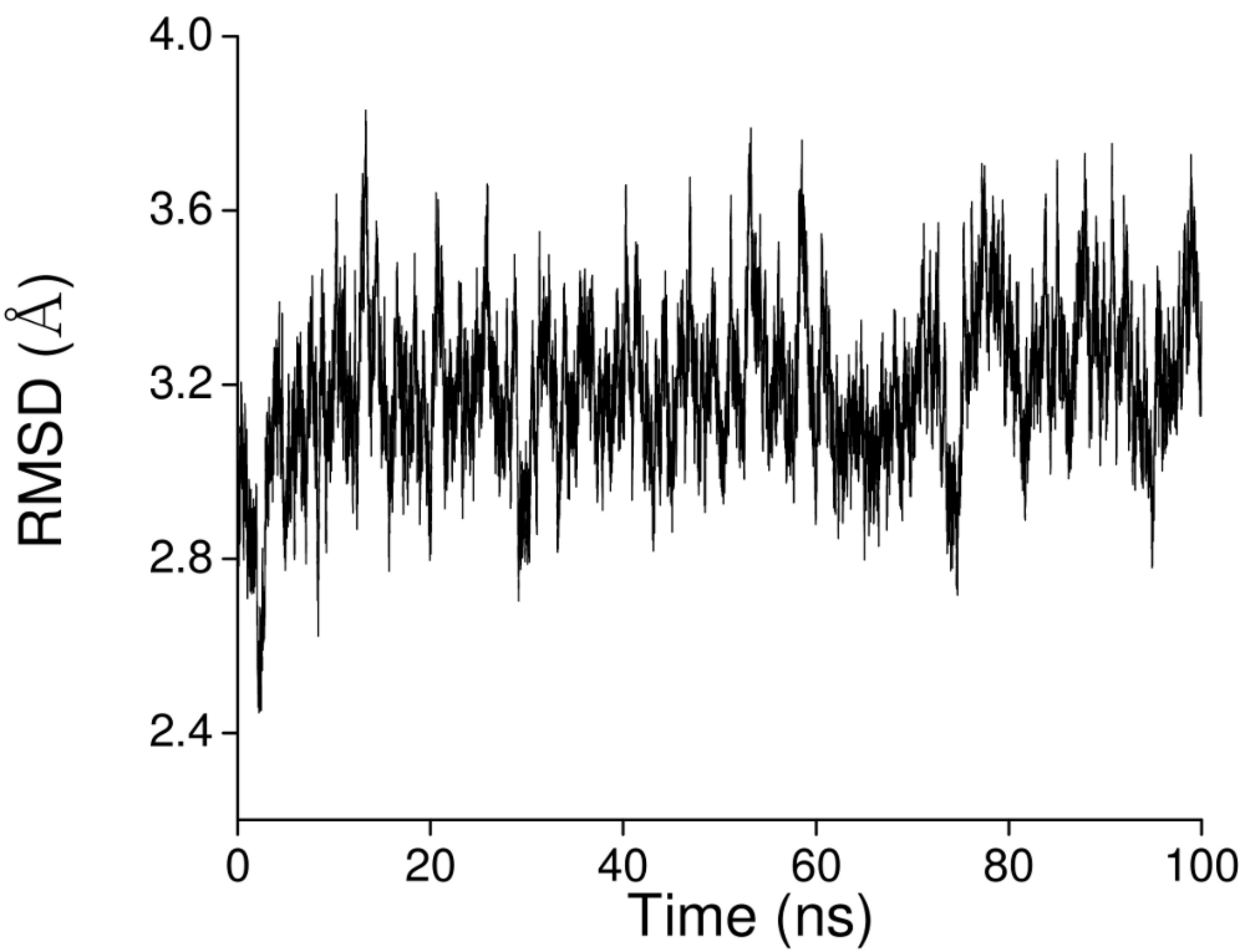

Supplement: Figure S3 — DNA phosphorous atom RMSD versus simulation time for intact nucleosome. (PDF) [file pcbi.1002279.s005.pdf]

Number of H-bonds

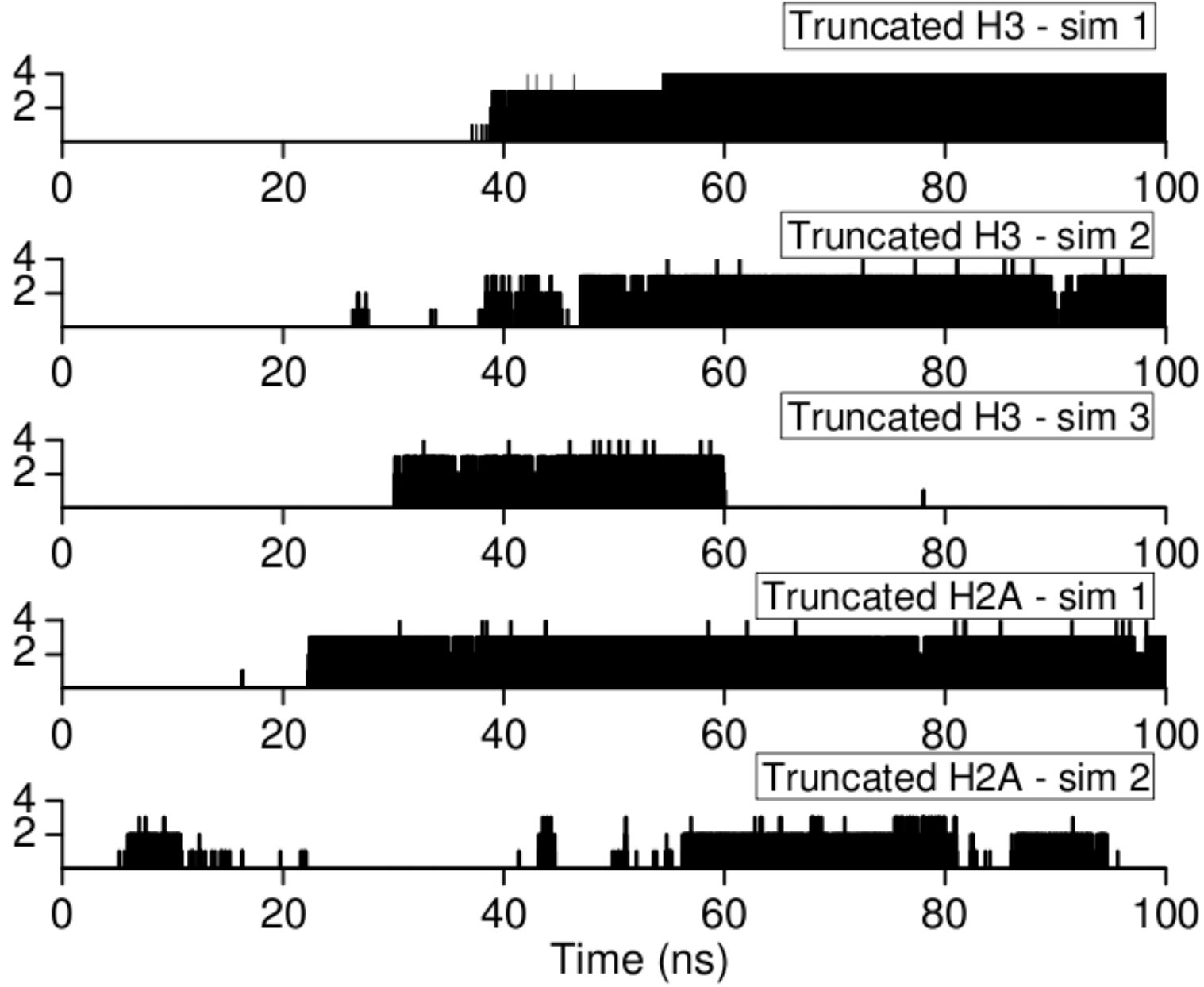

Supplement: Figure S4 — Number of hydrogen bonds between Arg88 of H2A(2) and Glu105 of H3(1) or Ala135 of H3(2) as a function of time for tail-truncated nucleosome simulations. In the H2A tail-truncated nucleosome simulation number 2 no hydrogen bond was formed between Arg88 and Glu105. (PDF) [file pcbi.1002279.s006.pdf]

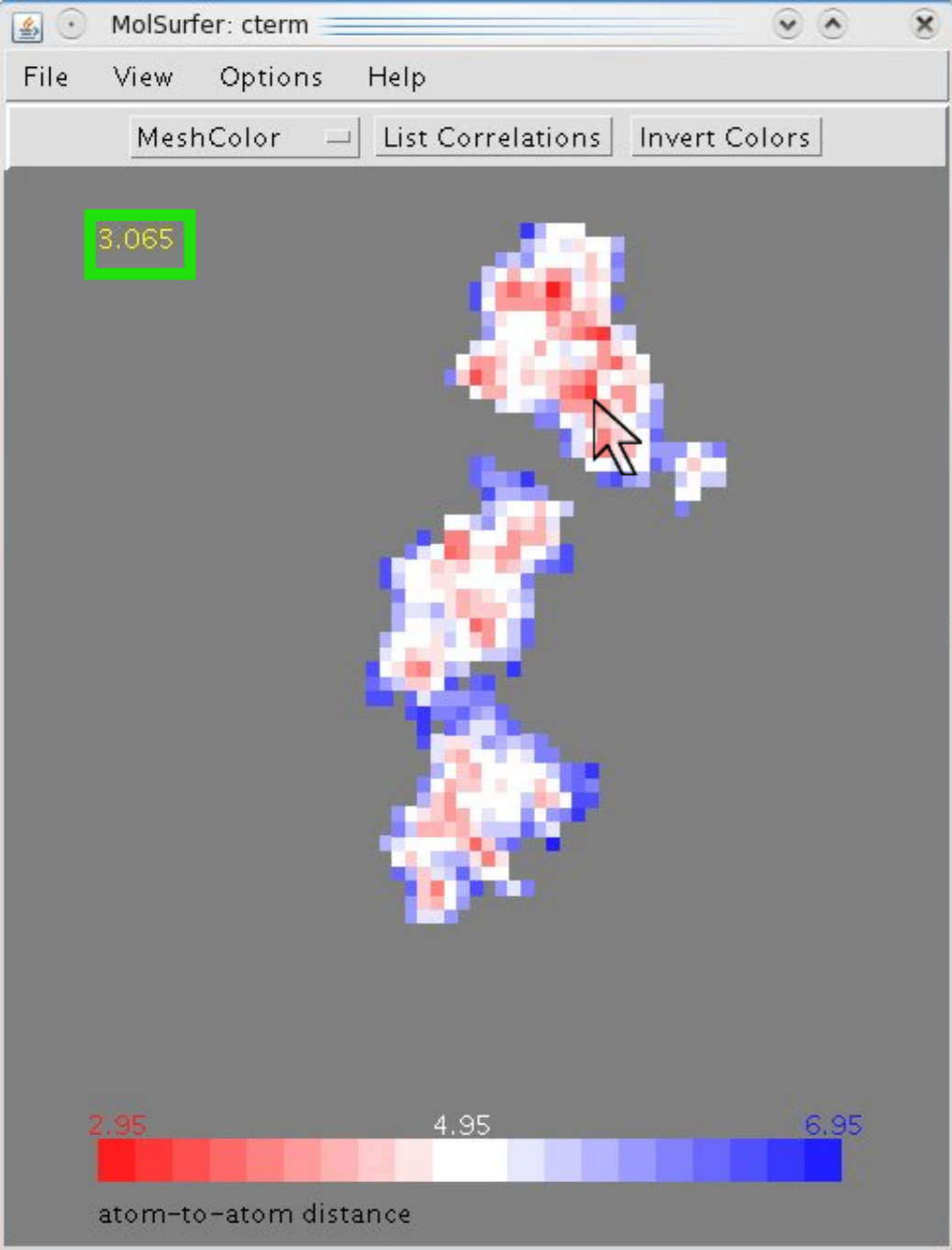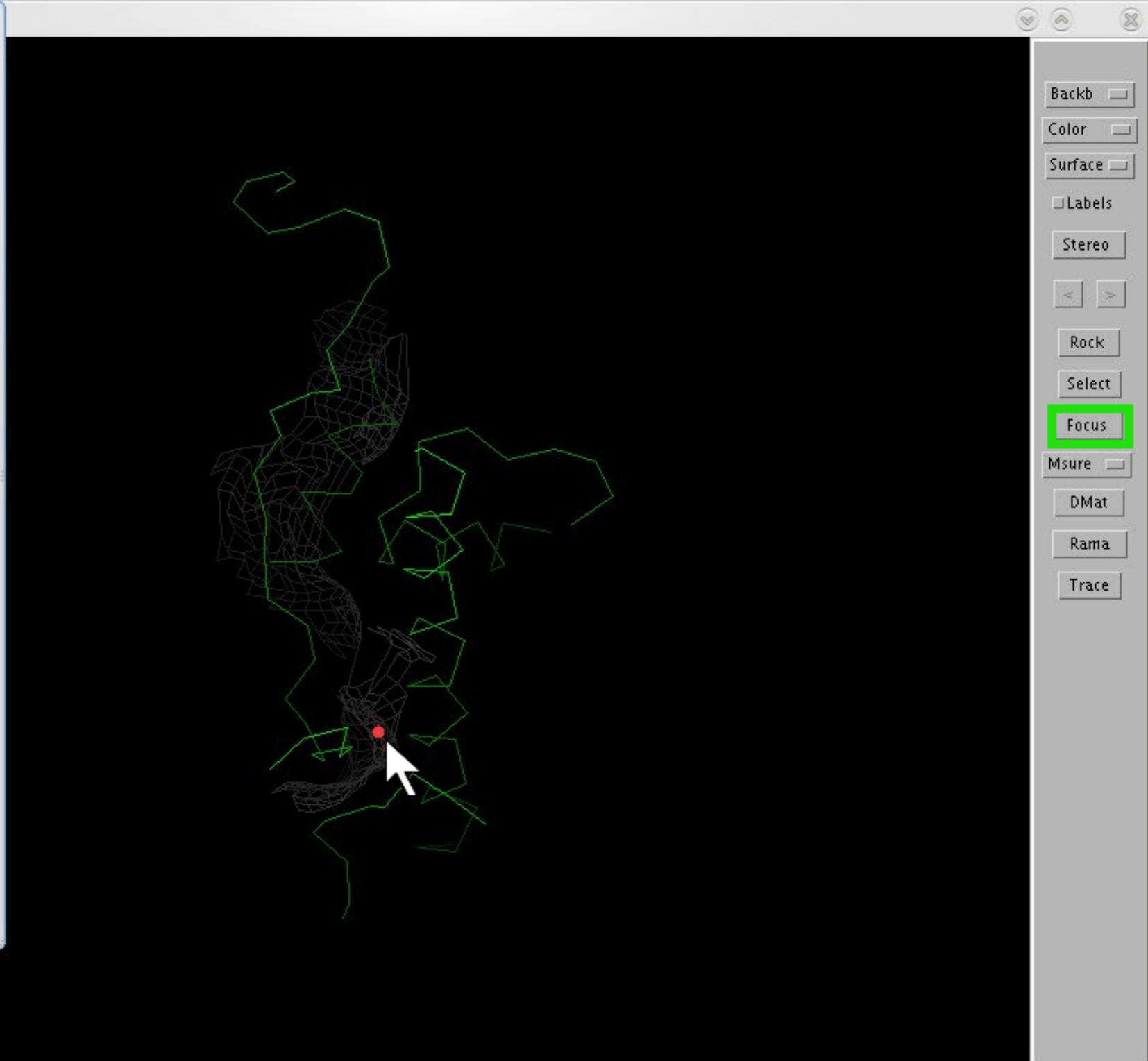

Supplement: Figure S5 — The 2D contact map of the H2A docking domain with the WebMol interface for viewing the docking domain and its surrounding in 3D. In the WebMol interface atoms are shown in backbone representation. The interface between the H2A docking domain and its surrounding appears as a mesh. (PDF) [file pcbi.1002279.s007.pdf]
