# Supplementary material for: Role of Histone Tails in Structural Stability of the Nucleosome
Source: PLoS Comput Biol. 2011 Dec 15;7(12):e1002279. doi: 10.1371/journal.pcbi.1002279 (PMC3240580; doi:10.1371/journal.pcbi.1002279)
Supplement: Table S1 — Comparison between 20 ns [19] and 100 ns nucleosome trajectories. (PDF) [file pcbi.1002279.s008.pdf]

*Supplementary Table 1: Comparison between 20 ns [Ref. 19] and 100 ns nucleosome trajectories*

| | | 20 ns | 100 ns |
|---|---|---|---|
| Intact nucleosome simulation | | | |
| Backbone RMSD | H3(1)<br>H3(2) | ~ 1.2 Å | 1.31 ± 0.13 Å<br>1.32 ± 0.13 Å |
| | H4(1)<br>H4(2) | ~ 0.8 Å | 1.32 ± 0.16 Å<br>1.15 ± 0.19 Å |
| | H2A(1)<br>H2A(2) | ~ 2.1 Å | 1.41 ± 0.16 Å<br>1.50 ± 0.19 Å |
| | H2B(1)<br>H2B(2) | ~ 0.9 Å | 1.56 ± 0.24 Å<br>1.37 ± 0.17 Å |
| Conservation of protein secondary structure | | x | α-helics formation in H3 and H2B tail during simulation |
| DNA RMSD | | ~ 2.5 Å | 3.2 ± 0.17 Å |
| DNA backbone conformation | | B1 type | B1 type |
| | | | |
| Tail-truncated simulations | | | |
| Structural changes in the H2A2α3 domain | | x | Structural changes in the sidechain hydrogen bonding Arg81 and Arg88 are observed in H3 and H2A tail-truncated simulations |
| Change of interaction between the H2A docking domain and contact residues | | x | Interaction changes observed in H3 and H2A tail-truncated simulations. |
