# Supplementary material for: Role of Histone Tails in Structural Stability of the Nucleosome
Source: PLoS Comput Biol. 2011 Dec 15;7(12):e1002279. doi: 10.1371/journal.pcbi.1002279 (PMC3240580; doi:10.1371/journal.pcbi.1002279)
Supplement: Table S2 — Interaction change in histone monomers with respect to key findings. (PDF) [file pcbi.1002279.s009.pdf]

Supplementary Table 2: Interaction change in histone monomers with respect to key findings

Simulation type	Simulation number	Observed structural change in histone	Monomer 1	Monomer 2
Intact nucleosome	1	Formation of α-helix in H3 tail	√	x
H3 tail truncated nucleosome	1	Change of interaction in Arg81 of H2A	x	√
	2		x	√
	3		x	x
	1	Change of interaction of Arg88 of H2A	x	√
	2		x	√
	3		x	√
	1	Destabilization of H2A docking domain contacts with closely lying residues	x	√
	2		x	√
	3		x	√
H2A tail truncated nucleosome	1	Change of interaction of Arg88 of H2A	√	√
	2		√	√
	1	Destabilization of H2A docking domain contacts with closely lying residues	√	√
	2		√	x
